# Phosphatase PHLPP2 regulates the cellular response to metabolic stress through AMPK

**DOI:** 10.1038/s41419-021-04196-4

**Published:** 2021-10-04

**Authors:** Yan Yan, Karl N. Krecke, Aditi S. Bapat, Tingyuan Yang, Michael W. Lopresti, Douglas G. Mashek, Ameeta Kelekar

**Affiliations:** 1 Department of Pharmacology, University of Minnesota, Minneapolis, MN 55455 USA; 2 Department of Laboratory Medicine and Pathology, University of Minnesota, Minneapolis, MN 55455 USA; 3 Department of Biochemistry, Molecular Biology and Biophysics, University of Minnesota, Minneapolis, MN 55455 USA; 4 Department of Medicine, Division of Diabetes, Endocrinology, and Metabolism, University of Minnesota, Minneapolis, MN 55455 USA; 5 Masonic Cancer Center, University of Minnesota, Minneapolis, MN 55455 USA; 6 Present Address: Department of Surgery, University of Miami, Miami, FL 33136 USA

**Keywords:** Cancer metabolism, Stress signalling

## Abstract

PHLPP2 is a member of the PHLPP family of phosphatases, known to suppress cell growth by inhibiting proliferation or promoting apoptosis. Oncogenic kinases Akt, S6K, and PKC, and pro-apoptotic kinase Mst1, have been recognized as functional targets of the PHLPP family. However, we observed that, in T-leukemia cells subjected to metabolic stress from glucose limitation, PHLPP2 specifically targets the energy-sensing AMP-activated protein kinase, pAMPK, rather than Akt or S6K. PHLPP2 dephosphorylates pAMPK in several other human cancer cells as well. PHLPP2 and pAMPK interact with each other, and the pleckstrin homology (PH) domain on PHLPP2 is required for their interaction, for dephosphorylating and inactivating AMPK, and for the apoptotic response of the leukemia cells to glucose limitation. Silencing PHLPP2 protein expression prolongs the survival of leukemia cells subjected to severe glucose limitation by promoting a switch to AMPK-mediated fatty acid oxidation for energy generation. Thus, this study reveals a novel role for PHLPP2 in suppressing a survival response mediated through AMPK signaling. Given the multiple ways in which PHLPP phosphatases act to oppose survival signaling in cancers and the pivotal role played by AMPK in redox homeostasis via glucose and fatty acid metabolism, the revelation that AMPK is a target of PHLPP2 could lead to better therapeutics directed both at cancer and at metabolic diseases.

## Introduction

The PH-domain Leucine-Rich Repeat Protein Phosphatase, PHLPP2, is a member of the PHLPP family of serine-threonine PP2C phosphatases, which function to suppress cell growth or to promote death. Originally identified in a search for a phosphatase that targeted Akt-like PH domain-containing proteins, PHLPPs have emerged as major regulators of the PI3K/Akt signaling cascade [1–3]. Additional PHLPP targets, including Protein Kinase C (PKC), S6 Kinase 1 (S6K1), and Mammalian STE20-like protein 1 (Mst1), have since been identified [1, 4–6]. Dephosphorylation of oncogenic kinases Akt, S6K1, and PKC by PHLPPs, terminates downstream signaling and leads to growth suppression, whereas dephosphorylation of Mst1 activates its apoptotic function. In keeping with their tumor suppressor role, *PHLPP* genes are deleted in many cancers while protein expression is downregulated in others [7, 8]. However, PHLPPs are also stably expressed in some hematological malignancies [7], suggesting their enzymatic activity is subject to posttranslational control.

Nutrient limitation effectively increases metabolic stress by lowering intracellular energy levels. Low ATP levels and increased AMP/ATP ratios rapidly upregulate AMP-activated protein kinase (AMPK), a key energy-sensing kinase and master regulator [9]. AMPK, a heterotrimeric protein comprising one catalytic α subunit, one β, and one γ regulatory subunit, functions to restore homeostasis when cellular energy is low by activating glucose and fatty acid uptake and oxidation [10]. Interaction of AMP with the catalytic subunit causes a conformational change that promotes phosphorylation of threonine 172 (Thr^172^) and activates the kinase [11]. Phosphorylation of its downstream targets, such as Acetyl-CoA Carboxylase (ACC), ULK1, and TSC2 [12, 13] drives catabolic pathways, primarily fatty acid oxidation (FAO) and autophagy, resulting in increased ATP production while shutting down ATP-consuming fatty acid and protein synthesis [14]. Thus, the AMPK kinase/AMPK phosphatase axis is important for controlling AMPK activation and function, and for terminating AMPK signaling [15]. Upstream kinases that phosphorylate AMPK, the LKB1/STRAD/MO25 complex [16–18], and Ca^2+^-dependent CaMKKβ kinase, have been well described [19–21]. Few studies have also shown that PP2C family phosphatases can dephosphorylate AMPKα in vitro [22, 23].

**Fig. 1:**
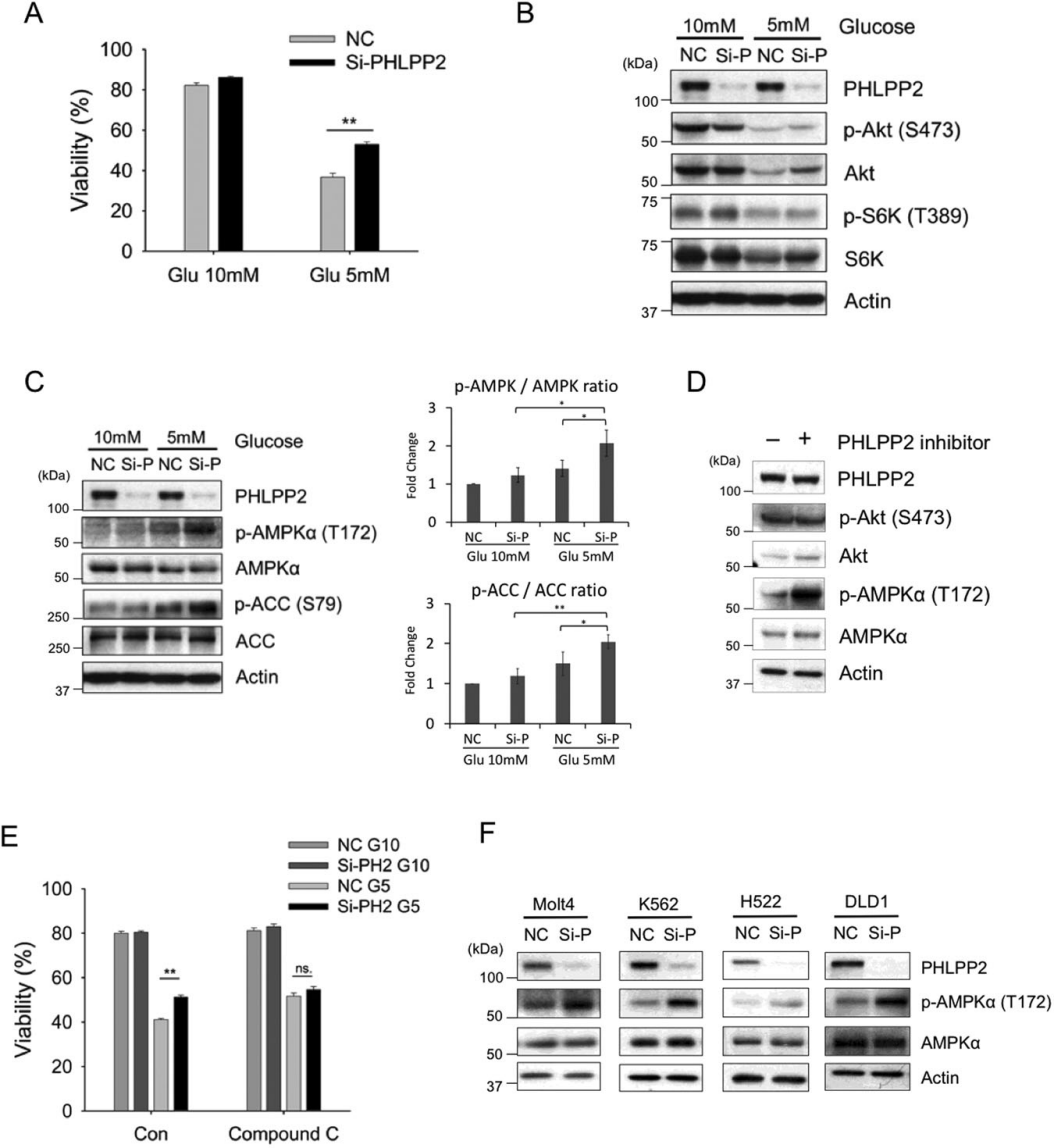
PHLPP2 suppresses cell survival under metabolic stress by targeting AMPK. **A** Jurkat cells were transfected with control siRNA (NC) or PHLPP2 siRNA (Si-PHLPP2) and, 24 h later, cultured for 4 days in 10 mM (high) or 5 mM (low) glucose medium. Cell viability was measured by flow cytometric analysis of Annexin V/PI uptake. Western blots of lysates from control siRNA (NC) or PHLPP2 siRNA (Si-P) transfected Jurkat cells, 3 days after growth in medium with 10 mM or 5 mM glucose showing phospho-Akt (S473), total Akt, phospho-S6K (T389), and total S6K protein expression (**B**), phosphorylation of AMPKα (T172) and downstream target, ACC (**C,** left), and averaged pAMPKα/AMPK and pACC/ACC ratios scanned from three independent experiments (**C**, right). **D** PHLPP2 inhibitor increases phosphorylation of AMPKα. Western blot showing phospho-AMPKα and total AMPKα in lysates of Jurkat cells grown in the presence of a PHLPP2 inhibitor (20 μM) for 72 h. **E** AMPK inhibitor, Compound C, abrogates the pro-survival effect of PHLPP2 loss under metabolic stress. Control siRNA (NC) or PHLPP2 siRNA (Si-PH2) transfected Jurkat cells were cultured for 4 days in 10 mM or 5 mM glucose medium with or without 2 μM Compound C. Cell viability was determined by flow cytometric analysis of Annexin V/PI staining. **F** PHLPP2 silencing increases phospho-AMPKα levels in a variety of cancer cell lines. Immunoblots of lysates from Molt 4 (acute lymphocytic leukemia), K562 (chronic myeloid leukemia), H522 (non-small cell lung cancer), and DLD1 (colorectal adenocarcinoma) cells were transfected with control siRNA (NC) or PHLPP2 siRNA (Si-P). Seventy-two hours after transfection, protein levels were determined by WB. Two-way ANOVA analyses were used to compare experimental groups in (**A**, **E**).

Our interest in PHLPP2 stemmed from its identification as a component of a glucose-sensitive multi-protein particle in Jurkat T-ALL cells ([24] and unpublished). Evidence showing PHLPP2 was able to regulate the cellular response to energy stress induced by glucose withdrawal in Jurkat T-ALL cells (Fig. 1A) led us to ask whether PHLPP2, a PP2C phosphatase, targeted AMPK. The data demonstrate that PHLPP2 mediates the cellular response to metabolic stress not by dephosphorylating Akt or S6K but by targeting AMPK, making this conserved, energy-sensing kinase the newest addition to the small list of known PHLPP2 targets. Our studies suggest PHLPP2 deregulates homeostasis by preventing the utilization of fatty acid for energy production through an AMPK/ACC route when glucose supply is limited. In addition to offering a window into a new signaling pathway regulated by PHLPP2, the studies described here reveal that the targets of this, and other, PHLPP proteins, can vary with cell type and stress stimulus.

## Results

### PHLPP2 suppresses cell survival under metabolic stress by targeting AMPK

PHLPP family phosphatases function as tumor suppressors. Thus, their genes are often deleted, or poorly expressed, in cancers [25]. However, PHLPP proteins are also constitutively expressed in some cancers, including leukemia [7, 26]. We identified PHLPP2 in a cytosolic glucose-sensitive multiprotein particle from Jurkat T-ALL cells by MS analysis [24], which suggested a possible role for this phosphatase in glucose metabolism. A stable isotope tracer study, however, did not reveal significant differences in the enrichment of [^13^C]-labeled glycolytic or TCA cycle metabolites between [1,2-^13^C] D glucose-fed siPHLPP2 and control Jurkat cells, and only a small, albeit significant, enrichment in acetyl CoA, (Fig. S1B–D). Therefore, we asked whether PHLPP2 was required for the cellular response to glucose limitation. Jurkat cells, normally grown in 10 mM glucose, are sensitive to lower (2–5 mM) levels of glucose in the growth medium. PHLPP2 silencing imparted a survival advantage to Jurkat cells grown in 5 mM glucose (Fig. 1A). However, this survival response was not mediated via phosphorylation of two commonly known PHLPP2 targets, pAkt and pS6K (Fig. 1B).

Low glucose availability can alter intracellular AMP/ATP ratios and activate AMPK via T^172^ phosphorylation [9, 11], as well as that of its downstream targets, ACC and ULK1 [12, 13]. We observed a selective increase in AMPKα-pT^172^ phosphorylation, and as well as that of its substrate ACC in PHLPP2-silenced Jurkat cells under glucose stress (Fig. 1C), but no significant effects on pULK1 levels (Fig. S2). Exposure of the cells to a small molecule PHLPP2 inhibitor [27], again, did not affect pAkt, but increased AMPKα-pT^172^ levels (Fig. 1D). Thus, knock-down as well as pharmacological inhibition of PHLPP2, resulted in activation of AMPK and its downstream signaling pathways in T-ALL cells. Additionally, AMPK inhibitor Compound C [28, 29] abrogated the protective effect of PHLPP2 knockdown under glucose limitation (Fig. 1E). Together, the data point to PHLPP2 as a sensor of glucose availability and to AMPK as its target.

We checked whether PHLPP2 dephosphorylates AMPKα in other cancer cells. Figure 1F shows AMPKα-pT^172^ levels increased when PHLPP2 was silenced in Molt4 (acute lymphocytic leukemia) and K562 (chronic myeloid leukemia) cell lines, and in epithelial cancer lines H522 (non-small cell lung cancer) and DLD1 (colorectal adenocarcinoma). However, while the absence of PHLPP2 protected against cell death induced by metabolic stress in K562 and H522 cells, it had the opposite effect in Molt4 and DLD1 cells (Fig. S3A−D). This is not surprising, given recent studies showing activated AMPK can be tumor-suppressive or oncogenic, depending on the cellular context and on the α-subunit targeted [30–33].

Both PHLPP2 and PHLPP1 belong to the PHLPP family of phosphatases and are known to share targets [8]. To determine whether PHLPP1 also affected the viability and AMPK T^172^ phosphorylation we silenced both phosphatases, either singly or together, in Jurkat cells (Fig. S4A). Only PHLPP2 silencing increased cell viability under metabolic stress (Fig. S4B). Moreover, PHLPP1 knockdown had no effect on the phosphorylation of AMPKα (Fig. S4C). Thus, regulation of AMPKα phosphorylation and cell viability in human leukemia cells under glucose stress is unique to PHLPP2.

### PHLPP2 regulates phosphorylation and activation of AMPK in response to glucose availability

**Fig. 2:**
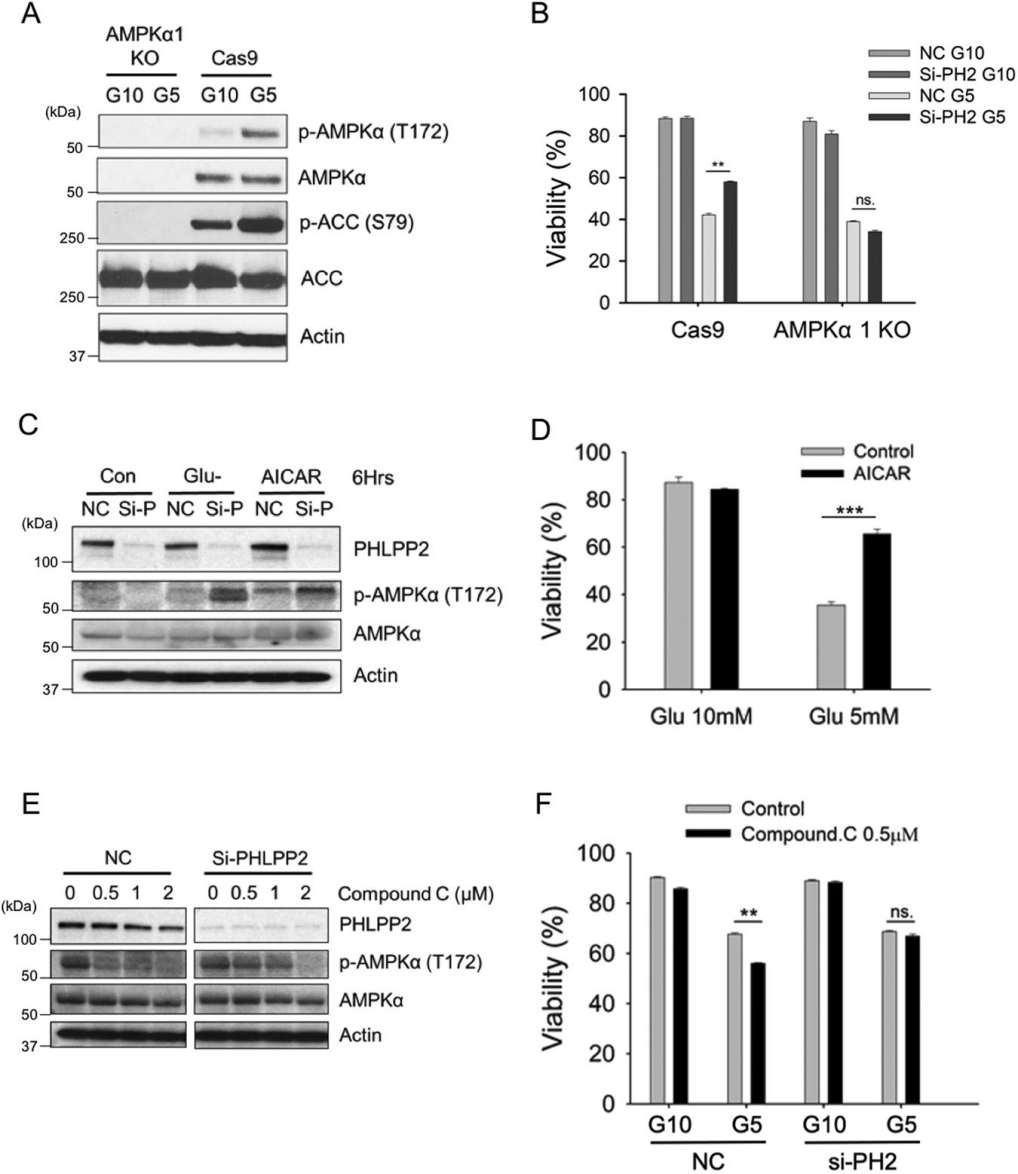
PHLPP2 regulates the phosphorylation and activation of AMPK in response to glucose availability. **A** AMPKα1 was knocked out in Jurkat cells through CRISPR-Cas9. AMPKα, phosphorylation of AMPKα (T172), and downstream target, ACC, were detected by WB after growing cells in a medium with 10 mM or 5 mM glucose for 3 days. **B** CRISPR-Cas9 control (Cas9) or AMPKα1 knockout (AMPKα1 KO) Jurkat cells, in which PHLPP2 was silenced, were cultured for 5 days in 10 mM or 5 mM glucose medium. Cell viability was measured by flow cytometric analysis of Annexin V/PI staining. **C** Both glucose deprivation and AICAR treatment promote phosphorylation of AMPKα in the absence of PHLPP2. WB of lysates from Jurkat cells cultured in glucose-free medium or treated with 0.5 mM AICAR for 6 h, 48 h after transfection with control siRNA (NC) or PHLPP2 siRNA (Si-P). **D** AICAR treatment prevents cell death under metabolic stress. Viability of Jurkat cells grown in 10 mM or 5 mM Glucose medium in the presence or absence of 0.2 mM AICAR for 3 days. **E** PHLPP2 silencing abrogates the inhibitory effect of Compound C on AMPKα. Jurkat cells transfected with control siRNA (NC) or PHLPP2 siRNA (Si-PHLPP2) were exposed 24 h later to 0.5, 1, 2 μM of Compound C for a period of 3 days. Protein levels were detected by WB. **F** The ability of Compound C to promote death under glucose stress is mitigated in the absence of PHLPP2. Jurkat cells, 24 h transfected, were cultured for 3 days in 10 mM or 5 mM glucose medium with or without 0.5 μM Compound C. Cell viability was measured by flow cytometric analysis of Annexin V/PI staining. Two-way ANOVA analyses were used to compare experimental groups in (**B**, **D**, **F**).

Jurkat cells express the α1 isoform of the AMPK-α subunit. We generated AMPKα1 knock out (KO) lines using a CRISPR-Cas9 approach followed by single-cell cloning. Figure 2A shows AMPKα1 expression in a Cas9 control cell lysate in comparison with a representative KO Jurkat cell clonal line, with concomitant loss of phosphorylated ACC, its downstream target. The protective effect of PHLPP2 silencing under low glucose conditions, as observed in Cas9 cells, was diminished in the AMPKα1 KO cells (Fig. 2B).

AMPK is phosphorylated and activated when AMP binds to its γ subunit [11]. AICAR (5-Aminoimidazole-4-carboxamide ribonucleotide), an AMP mimetic, also binds to the AMPKγ subunit, to induce phosphorylation of AMPKα and activate the kinase [34]. Both glucose deprivation and AICAR treatment increased phospho (p) AMPKα (T^172^) levels in the absence of PHLPP2 (Fig. 2C). AICAR also promoted survival of Jurkat cells in low glucose (Fig. 2D), consistent with the protective effects of PHLPP2 inhibition or loss, shown earlier (Fig. 1A).

Compound C was ineffective at inhibiting AMPK phosphorylation at concentrations below 2 μM, in the absence of PHLPP2 (Fig. 2E). Moreover, in a low glucose milieu, silencing PHLPP2 protected against the death-promoting effects of low (0.5 μM) concentrations of the AMPK inhibitor (Fig. 2F). These data further confirm that PHLPP2 targets, and inactivates, AMPK to promote cell death.

### PHLPP2 interacts with endogenous AMPK, with its PH domain required for interaction, phosphatase activity, and response to glucose limitation

**Fig. 3:**
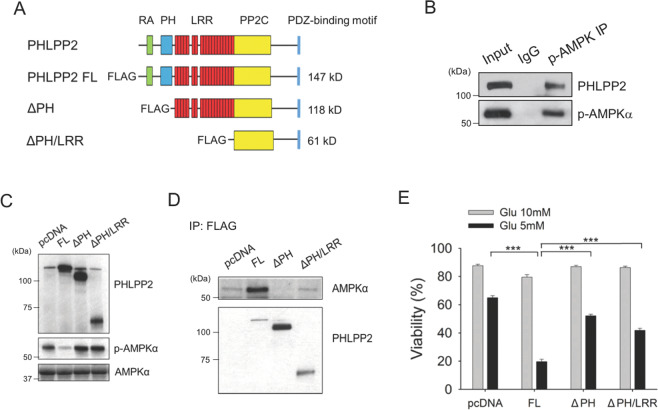
PHLPP2 interacts with endogenous AMPK and the PH domain is required for the interaction, phosphatase activity, and the response to glucose limitation. **A** Domain structure of FLAG-tagged full length and deletion mutants of human PHLPP2 used in transfection studies and phosphatase assays. RA Ras-Associated domain. PH Pleckstrin Homology domain. LRR Leucine-Rich Repeat domain. PP2C Protein Phosphatase 2C domain. **B** Endogenous PHLPP2 co-immunoprecipitates with phospho-AMPKα in Jurkat cell extracts. **C** Over-expression of full-length PHLPP2 induces dephosphorylation of AMPKα in Jurkat cells. WB of lysates from Jurkat cells transfected with empty vector, PHLPP2 full-length (FL), PH domain deleted (ΔPH), and PH/LRR domain deleted (ΔPH/LRR) constructs showing expression of transfected proteins and the phosphorylation of AMPKα (T172). **D** Full-length FLAG-tagged PHLPP2, but not the PH domain deleted mutant, interacts with AMPKα. Lysates from Jurkat cells transfected with the indicated construct were subjected to immunoprecipitation with anti-FLAG antibody 72 h after transfection. Immunoblots were probed for AMPKα and PHLPP2. **E** The PH domain is required for the death-promoting effect of over-expressed PHLPP2 under metabolic stress. Jurkat cells were transfected with the indicated constructs and 24 h later, cells were cultured in 10 mM or 5 mM glucose medium for 4 days and checked for viability by flow cytometric analysis of Annexin V/PI staining. Two-way ANOVA analysis were used to compare experimental groups.

PHLPP family proteins, including PHLPP2, harbor conserved functional domains, including a Ras-Association domain (RA), Pleckstrin Homology domain (PH), Leucine-Rich Repeat domain (LRR), PP2C phosphatase domain, and a PDZ-binding motif (Fig. 3A) [1, 25]. Since phosphatases are known to interact directly with their targets, we checked whether PHLPP2 could interact with pAMPK. Figure 3B shows that endogenous PHLPP2 co-immunoprecipitates with an anti-pAMPKα antibody. Previous studies have shown that PHLPP2 phosphatase activity is regulated by the PH domain and PDZ-binding motif [1, 4]. Endogenous AMPKα-pT^172^ levels were significantly reduced in Jurkat cells transiently over-expressing FLAG epitope-tagged full length PHLPP2, but not in cells expressing ΔPH or ΔPH/LRR deletion mutants (Fig. 3C). Anti-FLAG immunoprecipitations confirmed that deletion of the PH domain affected the interaction of endogenous AMPK with PHLPP2 (Fig. 3D). The ability of over-expressed full length PHLPP2 to promote apoptosis in Jurkat cells under low glucose conditions was also compromised in the absence of the PH domain (Fig. 3E).

AMPK was shown to be a target of PP2C phosphatases [22, 23], and we show here that targeting of T^172^ on AMPKα by PP2C phosphatase PHLPP2, disrupts the ability of AMPK to restore homeostasis.

### PHLPP2 dephosphorylates pAMPK in phosphatase assays in vitro

**Fig. 4:**
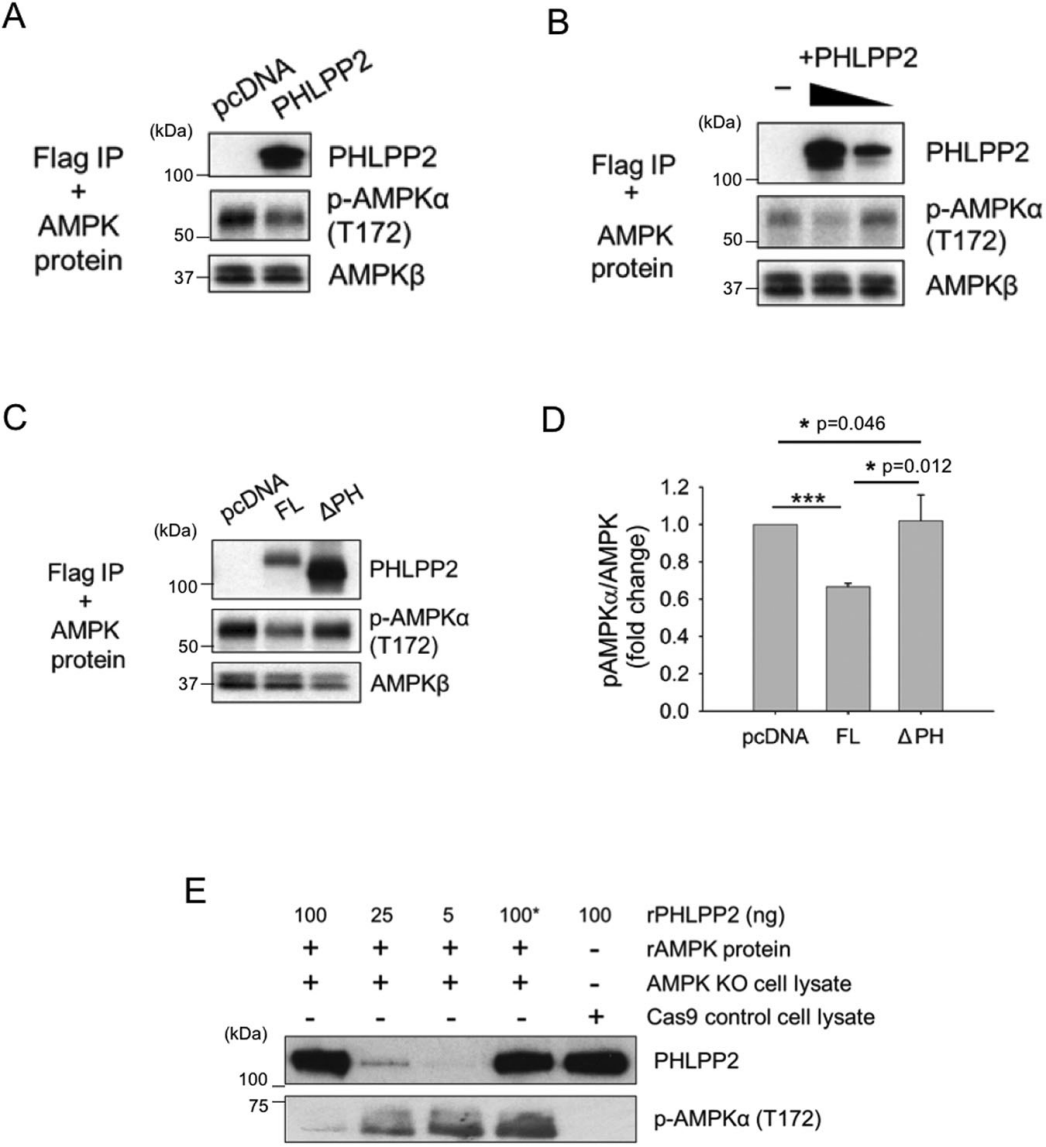
PHLPP2 dephosphorylates active recombinant (r)AMPK in phosphatase assays in vitro. **A** PHLPP2 dephosphorylates purified recombinant active p-AMPKα in vitro. Jurkat cells were transfected with an empty pcDNA vector or FLAG-PHLPP2 full-length construct. Seventy-two hours post transfection PHLPP2 proteins were immunoprecipitated and tested for phosphatase activity. The phosphorylation level of AMPKα and total AMPK levels (using AMPKβ as an indicator) were determined by WB. **B** PHLPP2 de-phosphorylates AMPKα in a dose-dependent manner. FLAG-PHLPP2 proteins that were immunoprecipitated from 400 μg or 200 μg cell lysate protein were included in the phosphatase assay. Phosphorylated AMPKα levels were determined by WB. AMPKβ expression was used as an indicator of total AMPK. **C** The PH domain is required for in vitro phosphatase activity PHLPP2 on AMPKα. Seventy-two hours after Jurkat cells were transfected with the indicated plasmids, expressed full length PHLPP2 or PH-deleted PHLPP2 pulled down with anti-FLAG agarose beads were tested in an in vitro phosphatase assay. **D** The graph shows the phosphorylation levels of AMPKα normalized to total AMPK level (checked by AMPKβ) after the in vitro assay shown in (**C**). pAMPKα and AMPKβ band densities were determined and the ratio of pAMPKα/AMPK in the PHLPP2 FL or ΔPH assay sets was normalized to that in the pcDNA control vector group. Data shown are the mean ± S.D from three independent experiments. The student’s two-tailed test was used for data analyses. **E** Phosphatase assay, in which indicated amounts of purified recombinant (r) PHLPP2 were incubated with recombinant p-AMPKα and assay buffer at 30 °C for 15 min in the presence of 10 μg AMPK-KO or Cas9 control cell lysate. * PHLPP2 was heat-inactivated prior to assay.

We next tested the ability of PHLPP2 to dephosphorylate recombinant active AMPKα in an in vitro phosphatase assay. Western blots in Fig. 4A, B show that transiently expressed FLAG-PHLPP2, immunoprecipitated from cell lysates, reduced phosphorylation in recombinant active AMPK-pT^172^ within 15 min of incubation, in a dose-dependent manner. The inability of PH domain-deleted PHLPP2 to dephosphorylate pAMPKα in the in vitro assay (Fig. 4C, D) could be attributed to the impaired interaction between ΔPH PHLPP2 and AMPK (Fig. 3D).

We also tested the ability of recombinant purified (r)PHLPP2 protein to dephosphorylate pAMPKα in vitro. Although we were unable to detect phosphatase activity with the recombinant protein in assay buffer alone (data not shown), rPHLPP2 significantly reduced levels of pAMPKα in the assay buffer in the presence of cellular protein lysate (Fig. 4E). The data suggest that PHLPP2 may require a regulatory intracellular scaffold protein or active co-factor to either stabilize its interaction with pAMPK or directly stimulate phosphatase activity. AMPK KO Jurkat cell lysates were the source of cellular protein in these experiments (endogenous pAMPK is undetectable in 10ug cellular protein from Cas9 controls). Figure S5 shows the results of an in vitro binding assay with the two recombinant proteins in the presence or absence of AMPK KO cell lysate. Data in Fig. 4E further confirm that PHLPP2 targets AMPK for dephosphorylation. Previous studies have demonstrated phosphatase activity of PHLPP proteins on Akt and PKC using PP2C domain peptides [1, 2], but this would be the first demonstration of PHLPP2 activity in vitro using the full-length purified recombinant phosphatase.

### PHLPP2 exerts its metabolic and apoptotic effects through an AMPK-ACC pathway

We showed increased phosphorylation of AMPK, and its downstream target ACC1/2, in glucose-deprived Jurkat cells, in the absence of PHLPP2 (Fig. 1C). Phosphorylation inhibits ACC activity, leading to increased catabolic FAO and decreased fatty acid synthesis (FAS) [35, 36]. The increase in pACC levels with PHLPP2 downregulation suggested that PHLPP2 contributed to metabolic stress-induced cell death through an AMPK-ACC pathway.

**Fig. 5:**
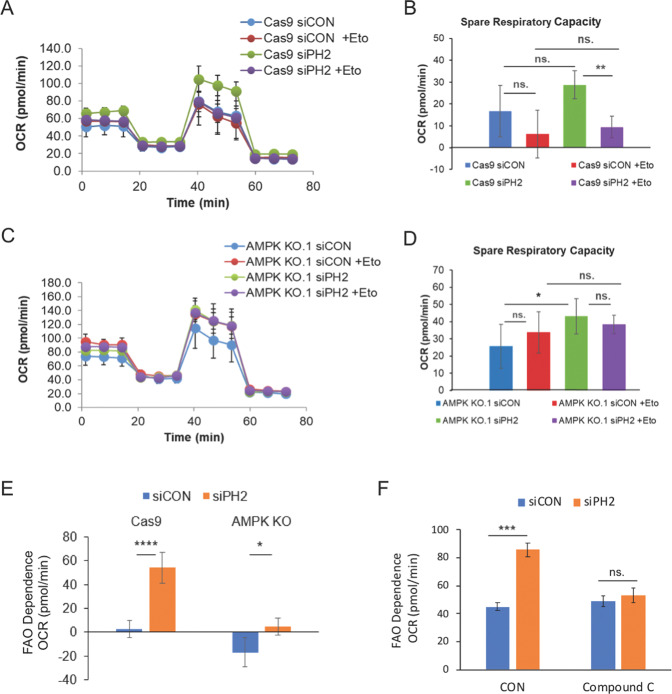
PHLPP2 exerts its metabolic and apoptotic effects through an AMPK-ACC pathway. The contribution of fatty acid beta-oxidation (FAO) to mitochondrial respiration was determined using a Seahorse FAO assay. Cells were cultured in nutrient-limited medium a day before the Seahorse assay and maintained in KHB medium for the duration of the assay (see “Methods”). Shown in the figure are mitochondrial oxygen consumption rates (**A**) and spare respiratory capacity (**B**) of siControl and siPHLPP2 expressing Jurkat cells in the presence or absence of 20 μM FAO inhibitor, Etomoxir (Eto). Mitochondrial oxygen consumption rates (**C**) and spare respiratory capacity (**D**) of control (Cas9) and AMPK knockout Jurkat cells transfected with siControl or siPHLPP2 RNA, in the presence or absence of Eto (*n* = 4−6). **E** Change in maximal oxygen consumption rates of siControl and siPHLPP2 Cas9 and AMPK knockout cells in the presence of FAO inhibitor, Eto. **F** FAO dependent OCR values in siControl cells or siPHLPP2 cells without (CON) or with Compound C treatment. An unpaired two-tailed Student’s t-test was used to compare groups. Data depict mean values (*n* = 3−7) and standard error; statistical significance was defined in the following manner— *p < 0.05, **p < 0.01, ***p < 0.001, ****p < 0.0001, and *p* values > 0.05 were considered not significant (ns).

To determine whether PHLPP2 influenced FAS we measured the incorporation of [^14^C]-acetate into triacylglycerols (TAG) and phospholipids (PL). Silencing PHLPP2 expression in Jurkat cells did not significantly affect *de novo* lipogenesis (Fig. S6A). To check dependence on FAO, we determined the sensitivity of the oxygen consumption rate (OCR) to FAO inhibition. Long-chain fatty acids are transported to the mitochondrial matrix through a carnitine palmitoyl transferase (CPT) system [37]. In mitochondria, fatty acid is oxidized to acetyl-CoA which enters the TCA cycle for further oxidation. Electrons derived from these steps move through the transport chain, generating energy for ATP synthesis. To determine whether PHLPP2 influenced fatty acid utilization through regulation of AMPK activity, under energy stress, we utilized the AMPK KO Jurkat cells and Cas9 controls described earlier (Fig. 2A). Control siRNA and PHLPP2-silenced Cas-9 cells were subjected to a Seahorse MitoStress test in nutrient-limited medium. PHLPP2 loss increased both maximum OCR and Spare Respiratory Capacity (SRC), but this was reversed in the presence of Etomoxir (ETO), a CPT1 inhibitor (Fig. 5A, B). Thus, cells lacking PHLPP2 showed greater dependence on FAO for respiration when metabolically stressed.

**Fig. 6:**
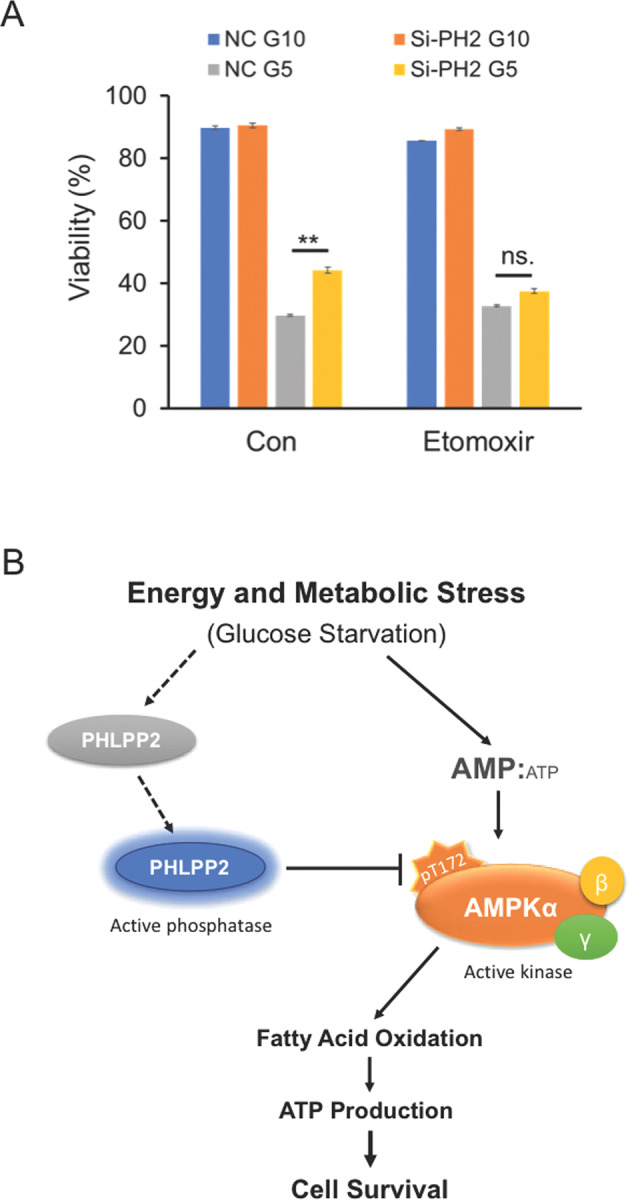
PHLPP2 regulation of the cellular response to metabolic stress is mediated by AMPK and fatty acid oxidation. **A** Pro-survival effects of PHLPP2 knockdown were reversed by FAO inhibition. Control siRNA (NC) or PHLPP2 siRNA (Si-PH2) transfected Jurkat cells, were cultured for 4 days (24 h post-transfection) in a medium containing either 10 mM or 5 mM glucose, in the presence or absence of 20 μM Eto, and analyzed for cell viability by flow cytometry. Two-way ANOVA analysis was used to compare experimental groups. **B** A simple model to illustrate how PHLPP2 functions as a tumor suppressor in cells undergoing metabolic stress. Under conditions of limited glucose availability, when AMP:ATP ratios are high, energy-sensing kinase AMPK is activated by phosphorylation and promotes fatty acid β-oxidation (FAO), enabling cells to generate energy and survive. Dephosphorylation of p-AMPKα at T^172^ by PHLPP2 inactivates the kinase and, subsequently, FAO, leading to cell death.

Etomoxir treatment did not significantly affect OCR and SRC in the knockout cells (Fig. 5C, D), which lacked an active FAO pathway, in keeping with the role of AMPK in fatty acid metabolism. Accordingly, PHLPP2 knockdown could no longer promote FAO in the AMPKα1 KO cells (Fig. 5E). Pharmacological inhibition of AMPK by Compound C reversed the FAO enhancement in PHLPP2 silenced cells (Figs. 5F, S6B, and S6C). Together, the data support a role for PHLPP2 in regulating FAO through an AMPK-ACC route during energy stress. The ability of PHLPP2 loss to protect against glucose deprivation-induced cell death (Figs. 1 and 2) is significantly reduced in the presence of FAO inhibition (Fig. 6A), further confirming the contribution of FAO to the protection imparted by PHLPP2 loss. The model in Fig. 6B summarizes our current understanding of how PHLPP2 prevents survival of metabolically stressed cells via regulation of AMPK activity and, thereby, fatty acid metabolism.

## Discussion

PHLPP phosphatases suppress cell survival either by inhibiting cell proliferation or promoting cell death [8]. Here we describe a novel growth inhibitory role for PHLPP2 in metabolically stressed cells that are mediated by the key energy sensor kinase, AMPK. Four PHLPP phosphatase targets, Akt, PKC, S6K1, and Mst1, had been identified since the initial discovery of the PHLPP family [1, 4–6]. Our studies show that in glucose-deprived T leukemia cells, activated PHLPP2 targets AMPK, rather than Akt or S6K.

In our Jurkat T leukemia model, loss of PHLPP2 promotes cell survival under glucose stress by increasing levels of AMPKα-pT^172^, effectively activating the kinase. AMPK activator AICAR also enables survival under these conditions, while inhibitor Compound C promotes death (Fig. 2D, F), suggesting an oncogenic role for AMPK in Jurkat cells. However, phosphorylation of the AMPKα subunit can also serve to inhibit cell viability and survival. In one study, AMPKα loss effectively accelerated Myc-driven lymphomagenesis [30] but other studies, showing AMPK promoting lung tumor cell survival and protecting leukemia-initiating cells during metabolic stress [32, 33], support an oncogenic role for the kinase. We observe that PHLPP2 also targets phospho-AMPK in solid tumor-derived cancer lines (Fig. 1F), although differences in the impact of PHLPP2 loss on their responses to glucose limitation (Fig. S3) suggest different roles for AMPK in the cell lines. These opposing roles for AMPK in cancer may depend on several factors, including cell type, context, and the expressed AMPK isoform. Emerging evidence suggests that the AMPKα2 subunit is a tumor suppressor, but the more commonly expressed AMPKα1 isoform is an oncoprotein [31].

Previous studies have shown that PHLPP1 and PHLPP2 phosphatases share targets [3]. One group recently demonstrated that PHLPP1 could regulate ER stress in mouse skeletal muscle myoblasts through dephosphorylation of AMPK, as well as known targets, such as Akt [38], although the study did not test a similar role for PHLPP2. We suggest that the two phosphatases exhibit lesser redundancy than previously believed, often showing context-dependent specificities, localization, and function. We previously identified PHLPP2 in a glucose-sensitive multi-protein particle in Jurkat T-ALL cells following mass-spectrometric analysis (unpublished). PHLPP2 functions as a sensor of glucose availability in primary tissue, such as pancreatic beta cells [39], and our study suggests it plays a similar role in some cancer cells. Other phosphatases that are activated by energy depletion and low fuel would be predicted to target AMPK, given its central role in energy homeostasis, and PP2A, PP2C, and PPM1E have been shown to inhibit its activity in cardiac and skeletal muscle, liver, and lung [40–44]. It would be of interest to determine whether their activation is cell type-specific or whether glucose limitation could also trigger one or more of these phosphatases to target AMPK in the T leukemia cell model described here, and whether there is physiologically relevant ‘crosstalk’ between phosphatases activated by the same trigger.

We have demonstrated phosphatase activity on AMPKα-pT^172^ using endogenous, transfected, and purified recombinant PHLPP2 (Fig. 4). PHLPP2 requires its PH domain for binding and dephos-phorylating AMPKα-pT^172^ in Jurkat cells in response to glucose stress. Furthermore, the inability of purified rPHLPP2 to dephos-phorylate recombinant pAMPK in an in vitro assay in the absence of cell extract (Fig. 4E) points to dependence on a regulatory factor or ‘scaffold’ protein to stabilize the interaction and/or trigger phosphatase activity. The phosphatase assays, using endogenous PHLPP2 immunoprecipitated from cell lysates (Fig. 4A−C), suggest that the cellular regulator may associate with PHLPP2. Other groups have shown that FKBP51, an immunophilin that interacts with both Akt and PHLPP2, serves as an adaptor molecule, providing a scaffold to facilitate PHLPP2 phosphatase activity [45, 46]. Future efforts will focus on identifying the putative cellular factor that could regulate PHLPP2 activity or its interaction with pAMPK.

AMPK exerts its oncogenic or tumor-suppressive function through regulation of glucose metabolism, redox homeostasis, and fatty acid synthesis/oxidation [36]. Increased phosphorylation of AMPKα in PHLPP2 silenced cells significantly increased phosphorylation of its downstream target Acetyl-CoA Carboxylase (Fig. 1C). Our studies point to a regulatory role for PHLPP2 in FAO, rather than FAS. When glucose supplies are limited, Jurkat cells switch to FAO to support ATP production and to survive, but only when PHLPP2 is not expressed. Thus, PHLPP2 promotes cell death under nutrient stress in part by preventing the switch to FAO. Our current understanding of how PHLPP2 modulates energy homeostasis through the regulation of AMPK and fatty acid metabolism is summarized in the simple model shown in Fig. 6B.

In conclusion, we offer evidence of a novel role for PHLPP2 in suppressing a survival response mediated through AMPK signaling, although the mechanism underlying its activation in response to metabolic stress remains elusive. Given the pivotal role of AMPK in coordinating cell growth and metabolism, new insights into the regulation of this key energy sensor by a tumor suppressor phosphatase could lead to better therapeutics directed not only at cancer but also at metabolic diseases.

## Materials and methods

### Reagents, chemicals, and antibodies

The antibodies directed against PHLPP1 (A304-029A) and PHLPP2 (PHLPPL, A300-661A) were purchased from Bethyl Laboratories (Montgomery, TX). Compound C was purchased from Sigma and AICAR was purchased from Cell Signaling Technology (Beverly, MA). Antibodies for P70S6K (9202), phospho-P70S6K (Thr389) (9205), Akt (9272), phospho-Akt (Ser473) (9271), ACC (3676), phospho-ACC Ser79 (11818), ULK1 (4776), phospho-ULK1 Ser757 (6888), AMPKβ (4150) were from Cell Signaling Technology. Antibodies for AMPKα (74461), phospho-AMPKα Thr172 (33524), and β-Actin (69879) were from Santa Cruz Biotechnology (Dallas, TX).

### Cell lines, cell culture, and transfection

Jurkat, Molt4, K562, H522, and DLD1 cells were grown in RPMI-1640, supplemented with 10% FBS, 2 mM L-glutamine, and non-essential amino acids. HEK293 cells were grown in DMEM with 10% FBS and 2 mM L-glutamine. All cells were supplemented with 100-units/mL penicillin and 100 ug/mL streptomycin and maintained in a humidified atmosphere of 5% CO_2_ at 37 °C. PHLPP2 full length or truncated constructs were transfected into Jurkat cells with a BioRad Electroporator, as described previously [24] or a Neon^TM^ Transfection System (Invitrogen) using the manufacturer’s protocol. Control, PHLPP1, and PHLPP2 siRNAs (GE Dharmacon, Lafayette, CO) were introduced into Jurkat, Molt4, K562, H522, and DLD1 cells using the Neon System.

### Glucose labeling experiments in cell culture

Live, healthy cells were recovered by Ficoll density gradient centrifugation within 24 h prior to the experiment and 10 million cells per sample were pelleted and washed in a glucose-free medium. Cells were resuspended at 1E6 cells/ml in complete glucose-free medium supplemented with 10% dialyzed FBS, NEAA, and 4mM L-glutamine for 1 h and starvation and supplemented with 10 mM [1,2-^13^C] labeled glucose for 24 h. Cells were then pelleted and washed once with cold PBS and resuspended in 100–200 μl −20 °C methanol, snap-frozen, and stored at −80 °C. Liquid chromatography/mass spectrometry (LC/MS) and gas chromatography/mass spectrometry (GC/MS) were used for the identification and quantification of labeled metabolites of all samples. [1,2-^13^C] glucose was purchased from Cambridge Isotopes (Tewksbury, MA USA).

### Western blotting and immunoprecipitation

For Western blotting, cells were lysed in RIPA buffer (50 mM Tris-HCl (pH 7.5), 150 mM NaCl, 0.5% v/v sodium deoxycholate, 1% v/v Nonidet P-40, 0.1% SDS) supplemented with protease and phosphatase inhibitor cocktails. Lysates were resolved by SDS-PAGE and transferred to nitrocellulose membrane. The blots were incubated with specific antibodies and chemiluminescent reactions were carried out using the ECL Plus kit (Amersham). Blots were stripped for reuse by washing for 30 min in TBS-T buffer (pH 2.5–3.0). For immunoprecipitation, cells were lysed in buffer A (10 mM HEPES, 0.015 mM MgCl_2_; 10 mM KCl; 0.05% IGEPAL) supplemented with protease and phosphatase inhibitors. Cell lysates were first cleared with protein A/G agarose beads and then incubated with either IgG or phospho-AMPKα antibodies overnight at 4 °C. The immunoprecipitated complex was recovered with Protein A/G beads and washed three times with Buffer A. Immunoprecipitated proteins were detected by western blotting as described above.

### Cell death assay

For cell viability studies, cells were collected under indicated conditions, washed once with cold PBS, and stained with Annexin V-FITC and Propidium iodide (PI) as described previously [24]. Flow cytometry data were analyzed using FlowJo software (Tree Star, Inc).

### Generation of AMPKα1 Knockout clones

PRKAA1, human AMPKα1 (4077757 seq G*U*U*GGCAAACAUGAAUUGAC) sgRNA was purchased from Synthego. The CRISPR/Cas9 electroporation mix, containing 100 uM PRKAA1 sgRNA in TE buffer and 1.0 mg/mL Cas9 mRNA (TriLink L7606), was incubated for 10 min, at room temperature, to allow RNP complexes to form. Jurkat cells, washed in PBS and resuspended in R buffer (Neon electroporation kit) at 2 × 10^7^ cells/mL, were added to the RNP solution and electroporated at 1325 V using 3 pulses at 10 ms intervals using a Neon Electroporator (ThermoFisher, Waltham, MA). The suspension was incubated for 48 h in an antibiotic-free culture medium before being transferred to a regular growth medium. Clonal populations, derived using limit dilution, were expanded and gene knockout was confirmed with TIDE [47] and Western blot analyses.

### In-vitro phosphatase assay

FLAG-tagged PHLPP2, immunoprecipitated from cell lysates, or purified recombinant PHLPP2 (Origene Technologies, Rockville, MD), was incubated with 25 ng of active recombinant AMPKα1β1γ2 (ThermoFisher) for 15 min at 30 °C in assay buffer containing 0.05 M Tris-HCL PH7.4, 1 mM DTT and 5 mM MnCl_2_. The reaction was terminated using Laemmli buffer without reducing agent. Phospho-AMPK α1 and total AMPK protein levels were determined through Western Blotting.

### De-novo lipogenesis assay

Media containing trace amounts of [1-^14^C] acetic acid were added to cells for 2 h. Subsequently, lipids were extracted and analyzed via TLC for incorporation of labeled acetic acid into neutral lipids as previously described [48].

### Seahorse FAO assay

Oxidation of fatty acids was measured using a modified version of the XF Cell Mito Stress Test (Seahorse/Agilent, Santa Clara, CA). Growth medium was replaced with substrate-limited medium (0.5 mM glucose, 1 mM GlutaMAX, 0.5 mM carnitine and 1% FBS) 24 h prior to the assay, 45 min ahead, cells were washed once with FAO assay medium (111 mM NaCl, 4.7 mM KCl, 1.25 mM CaCl_2_, 2 mM MgSO_4_, 1.2 mM NaH_2_PO_4_. 2.5 mM glucose, 0.5 mM carnitine and 5 mM HEPES, PH 7.4). Cells plated at 200,000 cells/well using CellTak (Corning, Oneonta, NY), were incubated in a non-CO_2_ incubator for 30–45 min at 37 °C. The assay cartridge was loaded with Stress Test inhibitors (final concentration: 2.5 μg/ml oligomycin, 2 μM FCCP, 2 μM rotenone/4 μM antimycin A), Etomoxir (Eto, final concentration 20 μM) or vehicle was added to cells, and the culture plate was incubated for 37 °C in a non-CO_2_ incubator for 15 min prior to starting the assay.

### Statistical analysis

Data show the average of at least three independent experiments. All averages are presented as mean ± s.d. Two-way ANOVA test and Student’s two-tailed test were used for data analyses: *p < 0.05; **p < 0.01; ***p < 0.001; ****p < 0.0001, and *p* values >0.05 were considered not significant (ns).

## Supplementary information

Supplemental Data

Author Contribution form

Reproducibility Checklist
