# Supplementary material for: Phosphatase PHLPP2 regulates the cellular response to metabolic stress through AMPK
Source: Cell Death Dis. 2021 Oct 4;12(10):904. doi: 10.1038/s41419-021-04196-4 (PMC8490465; doi:10.1038/s41419-021-04196-4)
Supplement: Supplementary file 1 — Supplemental Data [file 41419_2021_4196_MOESM1_ESM.pdf]

Supplemental Figure 1

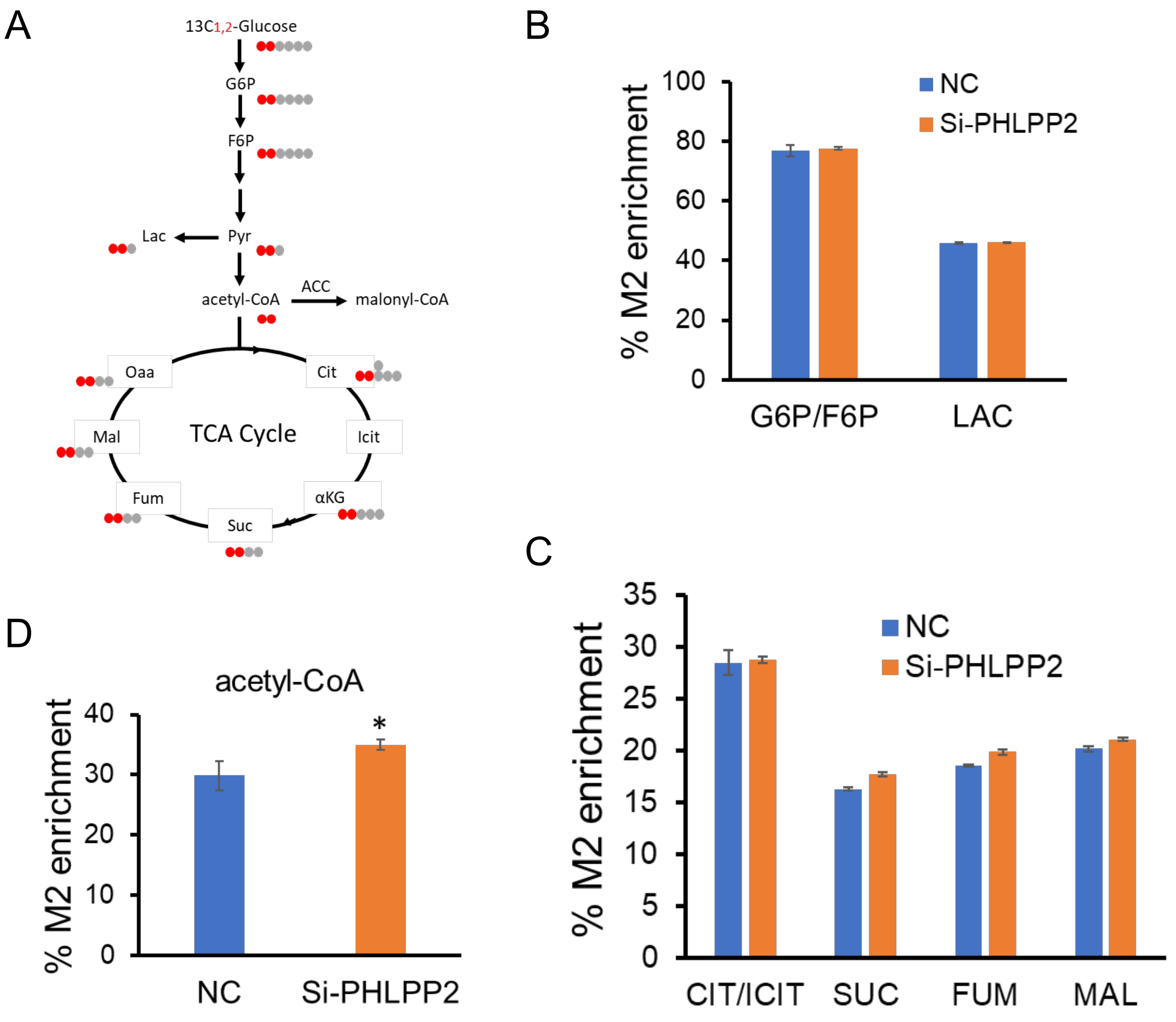

Supplemental Figure 1. PHLPP2 has little effect on glycolysis or TCA cycle. A. Schematic tracing the incorporation of [^{13}C] label from [1,2-^{13}C] glucose through glycolysis and the TCA cycle. **B.** Glycolysis metabolites labeled from [1,2-^{13}C] glucose following 24 h of labeling, shown as percentage of total metabolite measured using LC/MS. G6P, glucose-6-phosphate; F6P, fructose-6-phosphate; LAC, lactate. **C.** TCA metabolites labeled from glucose carbon measured via LC/MS following 24 h of labeling. CIT/ICIT, citrate/isocitrate; SUC, succinate; FUM, fumarate; MAL, malate. **D.** Acetyl-CoA labeled from glucose carbon measured via LC/MS following 24 h of labeling.

Supplemental Figure 2

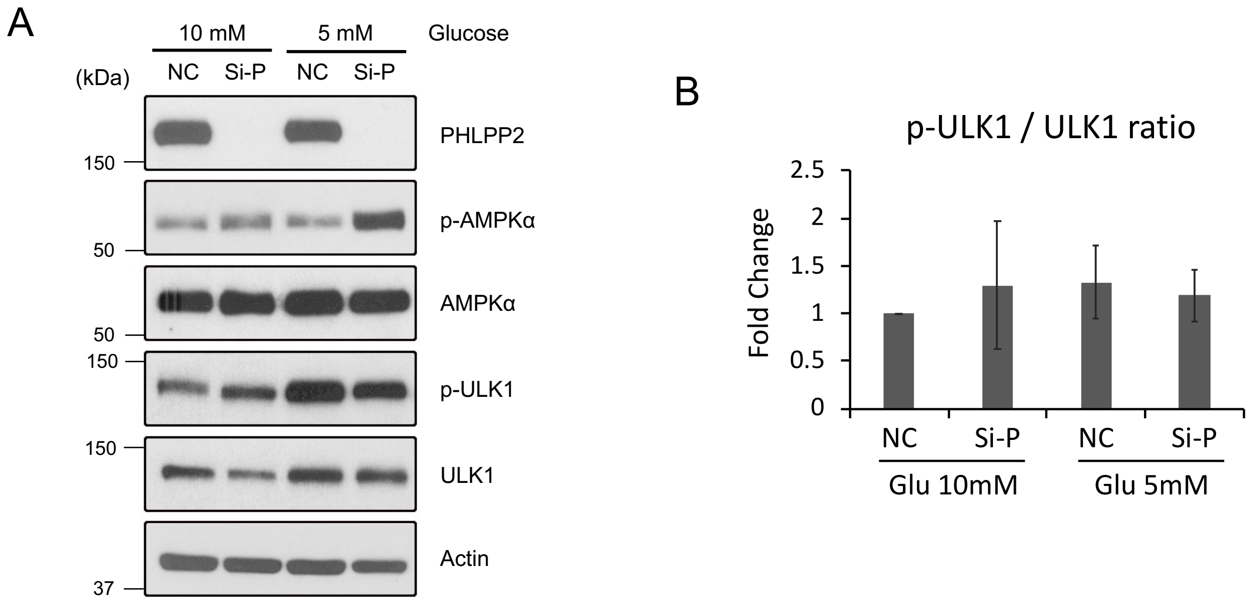

Supplemental Figure 2. PHLPP2 did not affect the phosphorylation of ULK1. A. Western blots of lysates from control siRNA (NC) or PHLPP2 siRNA (Si-P) transfected Jurkat cells, 3 days after growth in medium with 10 mM or 5 mM glucose showing phosphorylation of AMPKα (T172) and ULK1 (S757). **B.** The intensities of the bands in the western blots (A) were measured from three independent experiments. p-ULK1/ULK1 ratio was calculated by normalizing the intensity of p-ULK1 to that of ULK1.

Supplemental Figure 3

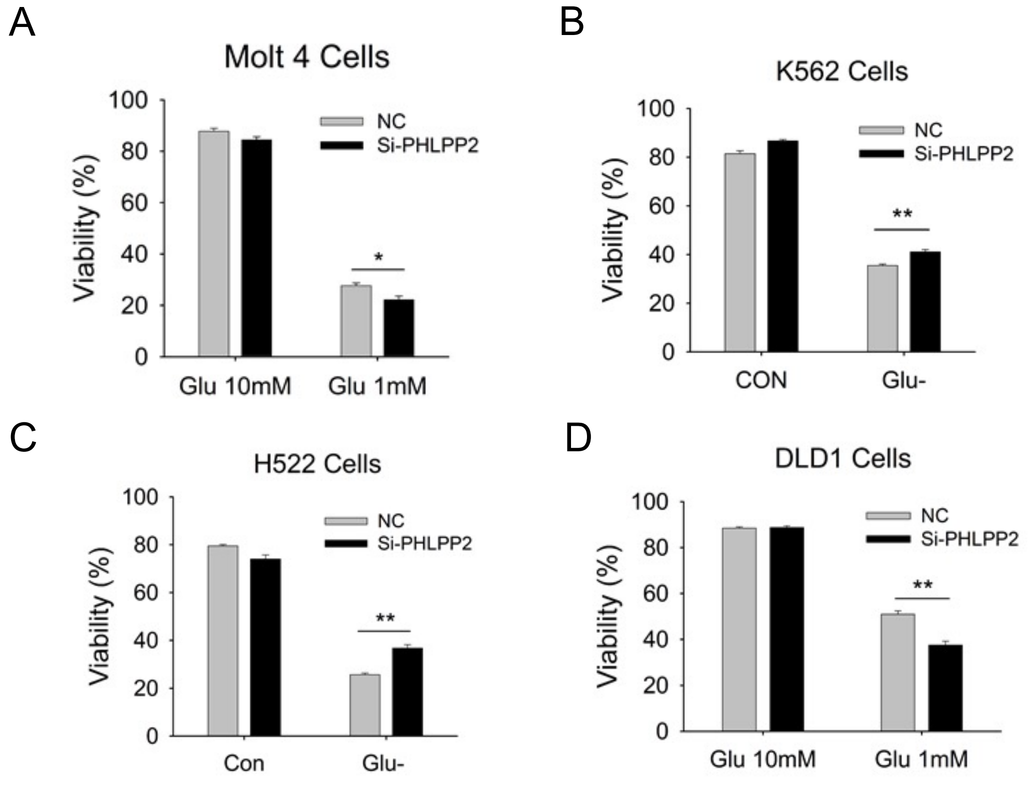

Supplemental Figure 3. PHLPP2 regulates the cellular response to metabolic stress in various tumor cell lines. (A) Molt 4 (acute lymphocytic leukemia), **(B)** K562 (chronic myeloid leukemia), **(C)** H522 (non-small cell lung cancer) and **(D)** DLD1 (colorectal adenocarcinoma) cells were transfected with control siRNA (NC) or PHLPP2 siRNA (Si-P). siRNA transfected cells were cultured in 10 mM or 1 mM glucose medium (Molt 4 and DLD1), 10 mM glucose medium or medium without glucose (K562 and H522) for 4 days. Cell viability was measured by flow cytometric analysis of Annexin V/PI staining.

Supplemental Figure 4

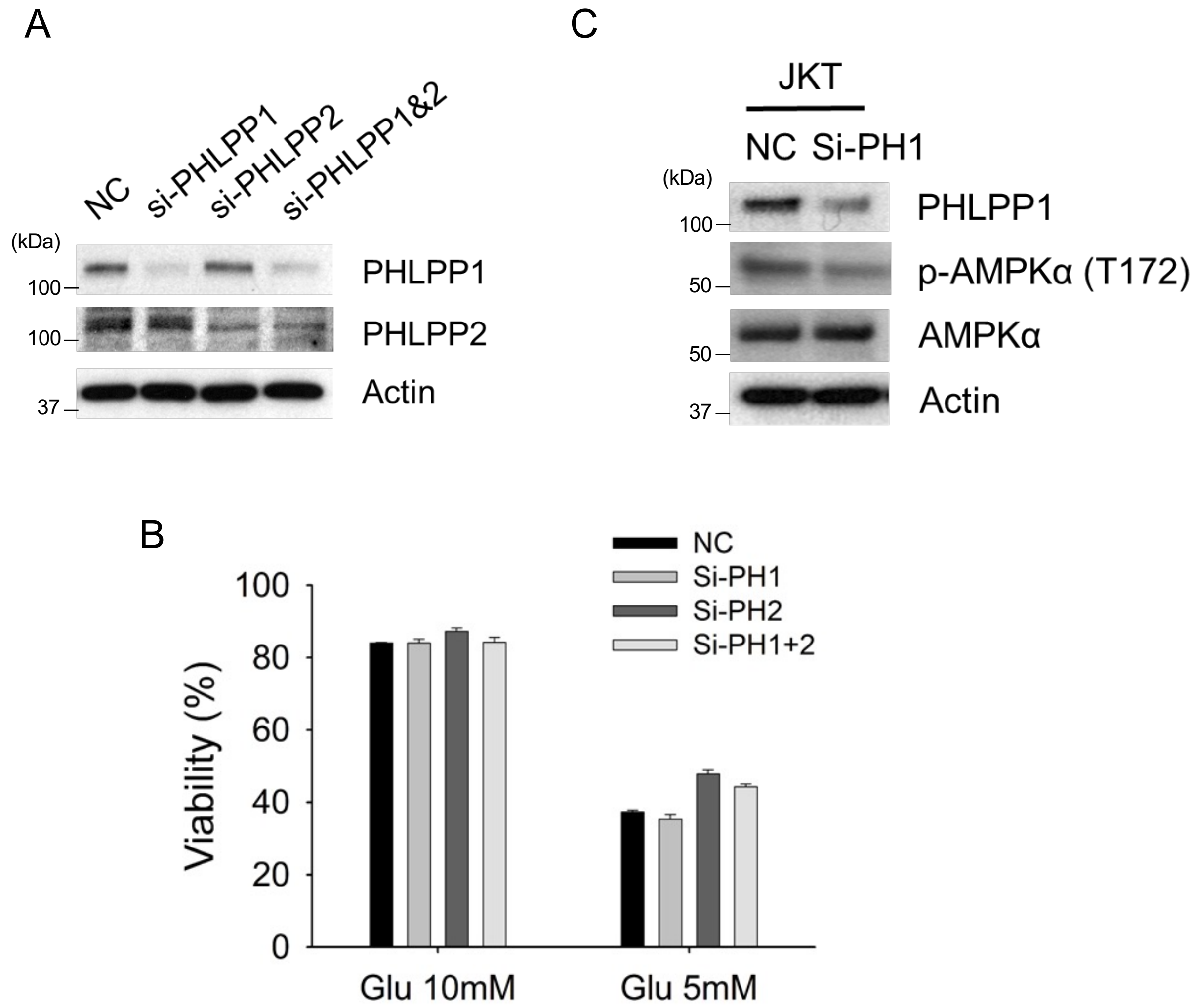

Supplemental Figure 4. PHLPP1 does not affect AMPK-phosphorylation or the cellular response to metabolic stress in Jurkat cells. A. PHLPP1 and PHLPP2 were silenced in Jurkat cells, either singly or together using siRNA. Jurkat cells were transfected with control siRNA (NC), PHLPP1 siRNA, PHLPP2 siRNA or both. 72 hours after transfection. Cells were collected to check the knockdown efficiency. **B.** Cell viability of Jurkat cells under low glucose conditions following PHLPP silencing. Jurkat cells were transfected with control siRNA (NC), PHLPP2 siRNA (Si-PH1), PHLPP2 siRNA (Si-PH2) or both (Si-PH1+2) and 24 hours after transfection the cells were cultured in medium with 10mM or 5mM glucose (Glu) for 4 days. Cell viability was measured by flow cytometric analysis of Annexin V/PI staining. **C.** PHLPP1 silencing does not affect AMPKα (T172) phosphorylation levels. Western blot shows pAMPKα (T172) levels in cells with PHLPP1 knocked down.

Supplemental Figure 5

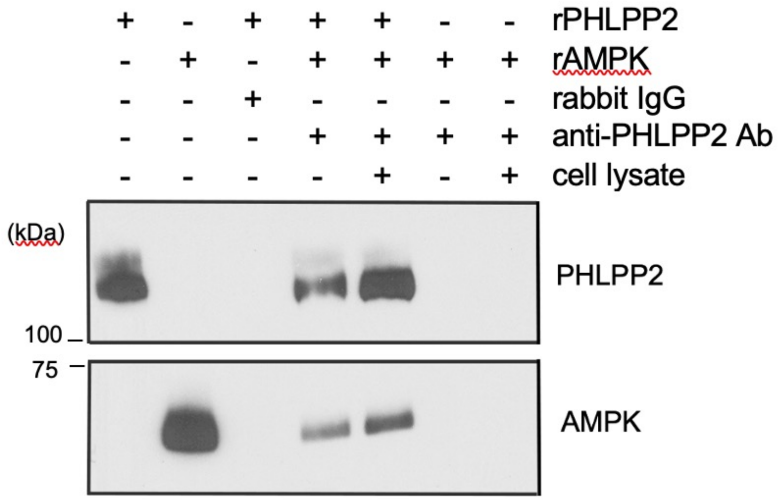

Supplemental Figure 5. Binding interaction between purified recombinant PHLPP2 and active rAMPK in vitro may be enhanced in the presence of cellular protein extract. PHLPP2 (100 ng) and recombinant p-AMPKα (40 ng) protein, incubated alone or together, in the presence or absence of AMPK KO Jurkat cell lysate (10 ug), at 25°C for 15 minutes in Buffer A, were immunoprecipitated using control IgG or anti-PHLPP2 antibodies, as indicated. Immunoprecipitated complexes were resolved by SDS-PAGE and immunoblotted for PHLPP2 and AMPK. Input lanes were loaded with 50 ng and 20 ng of rPHLPP2 and rAMPK, respectively.

Supplemental Figure 6

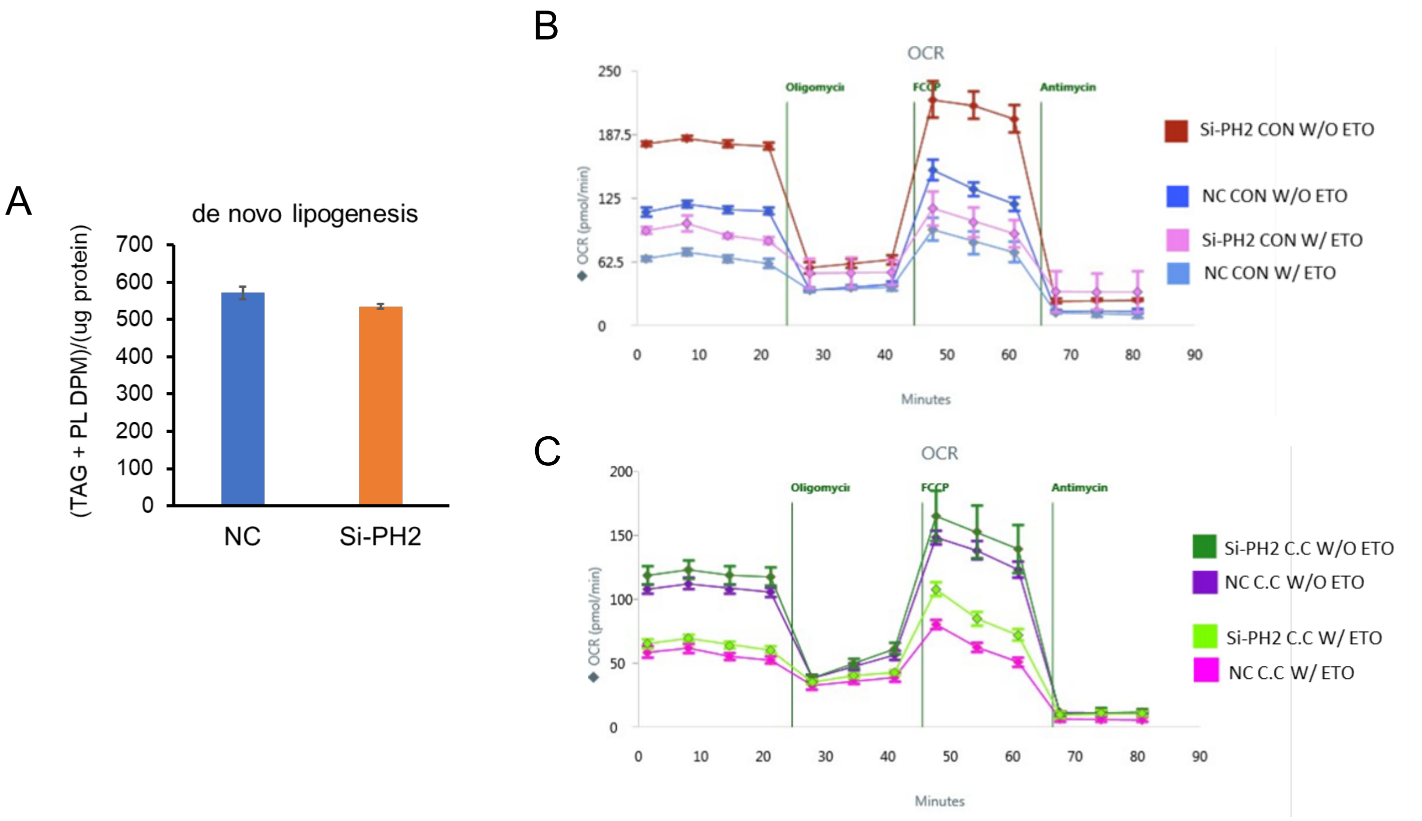

Supplemental Figure 6. Enhanced FAO observed in the absence of PHLPP2 was reversed by AMPK inhibitor Compound C. A. The incorporation of [1-^{14}C]-labelled acetate into triacylglycerols and phospholipids was measured to determine flux through *de novo* lipogenesis. **B.** Fatty acid oxidation was measured using a Seahorse FAO assay. The cells were cultured in nutrient limited medium a day before the assay and kept in KHB medium for the duration of the Seahorse assay (see methods). The drop in the OCR in the presence of 40μM Etomoxir is a measure of the contribution of FAO to the OCR. **C.** Jurkat cells were transfected with control siRNA (NC) or PHLPP2 siRNA (si-PH2) and 24 hours after transfection, treated with Compound C. Cells were cultured in nutrient-limited medium for another 24 hours and subjected to a Seahorse assay to measure FAO-dependent OCR.
