# Supplementary material for: Phosphatase PHLPP2 regulates the cellular response to metabolic stress through AMPK
Source: Cell Death Dis. 2021 Oct 4;12(10):904. doi: 10.1038/s41419-021-04196-4 (PMC8490465; doi:10.1038/s41419-021-04196-4)
Supplement: Supplementary file 2 — Author Contribution form [file 41419_2021_4196_MOESM2_ESM.pdf]

DECLARATION OF CONTRIBUTIONS TO ARTICLE

ADMC

Manuscript Number: CDDIS-20-4812R

Journal Name: *Cell Death & Disease* (the 'Journal')

Proposed Title of the Contribution:

Phosphatase PHLPP2 regulates the cellular response to metabolic stress through AMPK (the 'Contribution)

Author(s):

Yan Yan, Karl N. Krecke, Aditi S. Bapat, Tingyuan Yang, Michael Lopresti, Douglas Mashek and Ameeta Kelekar (the 'Authors')

For all *CDDis* articles, each person named as an author in the published version must be able to show he or she has contributed substantially to the article.

Authorship credit should be based on 1) substantial contributions to conception and design, acquisition of data, or analysis and interpretation of data; 2) drafting the article or revising it critically for important intellectual content; and 3) final approval of the version to be published. Authors should meet conditions 1, 2 and 3.

Any person who cannot be shown to have made a substantial contribution to the article cannot be listed as an author in the final version. The name of any person who is deemed to have made a minor contribution can, however, appear in the Acknowledgments section of the article.

Please complete the table below to indicate the contributions of all named authors to the manuscript.

Author Full Name:	Specification of Contribution to the Manuscript:
Yan Yan	Designed the study, performed experiments and data analysis and wrote the paper
Karl N. Krecke	Performed experiments, data analysis and edited the paper
Aditi S. Bapat	Carried out immunoprecipitation and western blot analysis and edited the paper
Tingyuan Yan	Carried out immunoprecipitation and western blot analysis
Michael Lopresti	Performed the in vitro lipogenesis assay in supplemental data
Douglas Mashek	Designed lipogenesis experiment and edited the paper
Ameeta Kelekar	Designed the study, analyzed data and wrote the paper

Please complete the table below to indicate the contributions of all named authors to the figures.

Figure 1:

YY 1A- F
KNK 1C
AK1A-F

Figure 2:

YY 2B-F
KNK 2A
ASB 2C
AK 2A-F

Figure 3:

YY 3B-E
TY 3B
AK 3A-E

Figure 4:

YY 4A-D
KNK 4E
AK 4A-E

Figure 5:

YY 5F
KNK 5A-E
AK 5A-F

Figure 6:

YY 6A
KNK 6B
ML 6B, Supplemental 6A
DM 6B, Supplemental 6A
AK 6AB

Signed for and on behalf of the Author(s):	Print Name:	Date:
Ameeta Kelekar	Ameeta Kelekar	July 22, 2021
